# Conflict of Interest: Are Iranian Breast Cancer Specialists Prone to it?

**DOI:** 10.31557/APJCP.2020.21.6.1653

**Published:** 2020-06

**Authors:** Amirpasha Ebrahimi, Sanaz Zand, Fahimeh Bagheri Amiri, Fahad Shahi, Ali Jafarian, Ahmad Kaviani

**Affiliations:** 1 *Department of Surgery, Tehran University of Medical Sciences (TUMS), Tehran, Iran.*; 2 *Department of Research, Kaviani Breast Diseases Institute(KBDI), Tehran, Iran.*; 3 *Urology and Nephrology Research Center, Shahid Beheshti University of Medical Sciences, Tehran, Iran.*; 4 *Department of Internal Medicine, Cancer Institute, Tehran University of Medical Sciences (TUMS), Tehran, Iran.*; 5 *Breast Diseases Research Center, Tehran University of Medical Sciences, Tehran, Iran. *; 6 *Department of Surgical Oncology, University de Montreal, Montreal, Canada.* ^*.*^

**Keywords:** Conflict of interest, ethics, breast cancer, physician, industry

## Abstract

**Introduction:**

Giving gifts is a common way to promote and encourage the use of products of trading companies and increase the patient referrals to diagnostic centers. The present study aimed to assess the practice of physicians of different (sub) specialties/educational levels engaged in breast cancer management in some conflict of interest (COI) situations in their relation with pharmaceutical companies and paraclinical centers.

**Methods:**

A self-administered online questionnaire including questions on demographic and professional information as well as the attitude of physicians toward COI by answering the questions in three different practical scenarios was developed. Respondents were asked to answer each question by selecting one of the five options: strongly agree, agree, undecided/neutral, disagree, and strongly disagree in their own practices as well as the same questions asking the same subject for what they think of the other physicians. Descriptive statistical analysis was used to report qualitative and quantitative variables.

**Result:**

The response rate was 66.24%. In general, physicians considered their performance better than that of other physicians in the situations asked. More than 90% stated that they would participate in the sponsorship congress for introducing new drugs. One fifth of the physicians stated that they would accept the 30% financial proposition for the referral of every single patient to other clinics. More than half of the physicians stated that they had considered the risks resulted from the COI for referring patients to private radiobiological centers.

**Conclusion:**

This study indicated that physicians in the field of breast cancer were at the risk of COI. Even within the medical field, there is not sufficient trust in the proper functioning of doctors in dealing with COI situations.

## Introduction

Both medicine and medical industries attempt to enhance the medical science, leading to many developments in the area of medicine. Although, there are some situations that may cause bias, as the main goal in the industry is to promote profitability, the medicine aims to improve the patient’s well-being. Both medical ethics and business ethics are legitimate, but they may be different (Coyle, 2002). The existence of a conflict between professional responsibility and personal interest may lead to conflict of interest (COI) (Stead, 2017). COI can manifest itself in several forms. It may include honorariums for writing or speaking about a company’s products, or referral to certain medical centers as well as receiving payment for participating in clinical research; the relationships of which may influence physicians’ attitude and practice (Kaviani et al., 2017). Many physicians maintain that the industry is not capable of influencing them; however, recent research demonstrates that gifts and hospitality can affect the judgment regarding the medical information and decisions on patient care (Coyle, 2002). Certainly, it should be noted that wherever there is a COI, there is not necessarily unethical behavior, and the judgement of the physician is important in this regard (Brody, 2010). In other words, existence of COI is not equal to bias occurrence; however, it can result in bias enhancement. The research supported by the industry may tend to the conclusions supporting the commerce; for example, publishing studies with positive results and publication bias, alterations in the prescriptions as well as promoting the use of surgical equipment, which may not be efficient or if unsafe, it could be dangerous to the patient. Based on the US Senator Grassley’s Physician Payments Sunshine Act (2009), any payment of more than $10 must be reported, and its data must be recorded on a searchable database (de Gara et al., 2013).

Many countries have strict rules for medical companies and physicians’ interrelations (Hajjar et al., 2017). The requirements of journals for authors to disclose any COI in clinical biomedical journals have increased in recent years (from one-sixth in 1997 to 99% in 2014) (Piper et al., 2018). In addition, some international medical companies have adopted ethical codes for regulating their interactions with medical vocations, preventing or removing the potential unethical actions and helping physicians and representatives to conform with the standard agreed for promotional activities (Hajjar et al., 2017). The studies conducted in countries with middle- and low-income, showed that the pharmaceutical company agents were meeting at least 90% of the physicians. This high percentage of interactions may influence the prescription habits and physicians’ professional behavior (Fadlallah et al., 2018). COI creates distrust in society, and its backlash affects not only the physicians, but also the holding institutes, their patients, supporting companies and all clinical research companies (Baim et al., 2007). As there does not appear to be any published study assessing the practice of physicians active in the breast cancer field in situations where COI may occur, this study was conducted with this purpose in mind.

## Materials and Methods

*The Study Design and Participants*

This study was conducted in the Surgery Department of Tehran University of Medical Sciences. The participants were physicians working in the field of diagnosis and treatment of breast cancer in private or public clinics. From April to October 2017, a self-administered questionnaire was sent to the physicians practicing in the breast cancer field. The questionnaire was developed using the discussion method in numerous gatherings of the experts in the field of medical ethics (Shahi et al., 2017). It was then finalized through consultation with a panel of ethics experts. The physicians were in different disciplines such as surgery, medical oncology, and radiation oncology. There was no concern for gender, age, work background, sector of medical practice, duration of medical experience, and having a background of medical ethics by attending the relative workshops. The individuals who studied medical ethics or graduates thereof were excluded from the study. 

*Data Gathering*

The questionnaire, which included demographic data, physicians’ attitude toward and practice in COI, was employed (Shahi et al., 2017). The practice questions included 5 presumed situations and practical scenarios. After reading each scenario, the physicians answered some questions on that situation about their own performance (response choices for part one: absolutely agree, agree to some extent, insufficient knowledge, disagree to some extent, absolutely disagree) and the participants’ opinion about the other physicians’ performance (part two: often, usually, no knowledge, sometimes, seldom). They could fill the questionnaire up either through an online system or through writing the answer in paper forms. The instruction on “how to fill up the questionnaire” was sent to the participants in advance.

*The Statistical Analysis*

The analysis was conducted by the SPSS version 23 software. The quantitative data (age, professional history work in medical ethics and professional activity in field of oncology) were changed to the dichotomous qualitative variable using median. In part one, the options ‘ agree to some extent’ and ‘I absolutely agree’ were considered a ‘positive’ answer. In part two, the options ‘often’ and ‘usually’ (from 50% to 100%) were considered a ‘positive’ answer to the respective performance and they addressed dichotomous quality variables. The qualitative data were reported as frequency and percentage. 

**Table 1:** Descriptive Analysis of the Participants’ answer to Scenarios, Including Answer to their own Practice and Attitude Toward other Physicians’ practice

Clinical		First part*		Second part**	
		Total	No of agree or strongly agree (%)	Total	No of doctors that have this practice more than 50%
The First Scenario					
Q-1	I do not allow advertisement of drugs in the medical environment.	91	65 (71.4%)	91	33 (36.3%)
Q-2	I accept the above-mentioned trip proposition.	91	30 (33.0%)	91	60 (65.9%)
Q-3	I always review the product of the company and prescribe it based on its advantages and preferences.	91	84 (92.3%)	91	55 (60.4%)
The Second Scenario					
Q-1	I will take part in the congress.	91	82 (90.1%)	91	77 (84.6%)
Q-2	According to what I have learned, I would prescribe the drug.	91	44 (48.4)	91	54 (59.3%)
Q-3	In case of attending the conference, I will research about sources of the presented drug very carefully, but I would be very cautious in prescribing the drug.	90	81 (90.0)	91	45 (49.5%)
Q-4	I would feel pessimistic about the medicine produced in a company, which has employed or partnered with international researches and would not prescribe the medicine.	90	19 (20.9)	91	12 (13.2%)
The Third Scenario					
Q-1	I will accept the 30% proposition.	91	18 (19.8%)	91	51 (56.0%)
Q-2	I will spend the amount taken for the poor patients or on developing the facilities of state clinical sectors.	91	33 (36.3%)	91	15 (16.5%)
Q-3	This will make me suspicious, and I will not use product B, unless in emergencies.	90	43 (47.8%)	91	13 (14.3%)
Q-4	I will investigate carefully product B and if there is no difference between products A and B, as before, I will prescribe both products.	90	74 (82.2%)	91	35 (38.5%)
Q-5	I will give feedback to company B on the unethical nature of the proposition.	91	69 (75.8%)	91	14 (15.4%)
The Forth Scenario					
Q-1	I will refer the patients to that center.	90	30 (33.3%)	91	52 (57.1%)
Q-2	I will accept the financial proposition, too.	91	20 (22.0%)	90	38 (42.2%)
Q-3	I will ask the manager of center A to consider a discount equal to the amount proposed for the financially poor patients referred by me and I will refer the patients.	90	56 (62.2%)	90	19 (21.1%)
Q-4	I will talk to the managing physician of center A about the unethical nature and unsuitability of their behavior.	91	64 (70.3%)	90	11 (12.2%)
The Fifth Scenario					
Q-1	Based on my inspections, I will refer the patient to the best center regarding 'quality and response time duration'.	91	81 (89.0%)	90	68 (75.6%)
Q-2	I will explain to the patient the reason for choosing the referred center.	90	89 (97.8%)	90	43 (47.8%)
Q-3	Notwithstanding the consideration of the patient's advantage, I will try to give the patient the right to choose.	91	87 (95.6%)	90	41 (45.6%)
Q-4	I will consider the dangers of COI and I will not refer the patient from a governmental center to a private one.	90	48 (53.3%)	90	27 (30.0%)

* Their own practice; ** They assumed the other physicians’ practice

## Results

In general, out of 157 invitations, 104 people (66.24%) returned the completed questionnaire. Thirteen participants had passed formal courses in medical ethics, which were excluded from the analysis. Finally, 91 physicians (55 males and 36 females) with the mean and median age of 44 were included in the study. The physicians stated that more than 16 patients with breast cancer referred to them weekly (with an average of 16.82 and a median of 10). Approximately 36% of the participants were surgeons. The number of fellowship graduates in different sub-specialties was 66 (72.5%). 

*Scenarios*

*The First Practical Scenario*

The adviser of a medical company X visits you to introduce their products and encourage you to prescribe or use them. The company offers to take you and your family on a one-week luxury trip to a European country in order to make you familiar with the product and participate you in a congress related to your field of specialty.

In response to this scenario, 71.4% of the physicians stated that ‘they would not allow the company to promote their products in the academic and medical environment’. However, they maintained that only 36.3% of other physicians would do the same. Although 33% of the physicians stated that they would accept the proposed trip, they assumed that 66% of the other physicians would accept it. More than 90% of them claimed that ‘they would study the company’s product as ever and would prescribe it based on scientific advantages and priorities’. However, they supposed that only 60.4% of other physicians would do the same. 

*The Second Clinical Case*

 The medical company X has organized a congress on its new medicine in a luxury hotel and invites you to participate in the congress to be familiarized with the medicine. In your personal primary review in the literature, you find out that the information indicating the effectiveness of the new medicine in the published studies. According to the seminar plan, the lecturer of the congress is an international authority who has a financial contract with the company and received a direct financial grant from the company.

Not only almost 90% of the individuals stated they would attend a congressional sponsorship event to be acquainted with the new drug, but also they believed that nearly 85% of the other their colleagues might accept attendance in sponsorship congress. 

Almost 50% of the physicians stated that based on what they had learned in the congress, they would prescribe the drug and believed that almost 60% of the other physicians would do so. Almost 90% of the physicians answered the question Q3 as ‘positive’; however, they believed that only 50% of the other physicians would do the same. Almost 21% of the physicians stated that ‘they were pessimistic about the produced medicine’, and they believed that almost 13% of the other physicians would be pessimistic.

*The Third Scenario*

The products of companies A and B are commonly used in your practice. Both products have proper quality and are approved. The manufacturer of product B gives a 30% discount per prescription and pays it to the prescribing physician.

Only 20% of the physicians stated that ‘they would accept the 30% financial proposition’, but they believed that 55% of the other physicians would accept the proposition. Only 36.3% of the physicians stated that ‘they would spend the taken amount for the poor patients or on developing the state clinical sector facilities’, but they assumed the same behavior for only 16.5% of the other physicians. Nearly half of the physicians answered question Q3 positively, but they believed that 14.3% of the other physicians would do so. More than 82% of the physicians maintained that ‘their action would be prescribing both products, if there were no difference between products A and B after experimenting with product B; however, they supposed this performance for only 38.5% of the other physicians. More than 75% of the physicians stated that ‘they would give the company B feedback on this unethical proposition, but supposed that this would be carried out by only 15.4% of the other physicians.

*The Forth Scenario*

Diagnostic center A is one of the good private centers with updated equipment in your city. An advisor from center A refers to you and states that he will pay you $50 per patient referred. You averagely visit 10 patients daily who should be referred to that institution for service. In addition, you are sure about the correct performance in the mentioned institution.

Only 33% of the physician stated that ‘they would refer the individuals to the same diagnosis centers’ and believed that almost 60% of the other physicians would do so. Almost 20% of the physicians claimed that they would accept a financial proposition, but they believed that more than 40% of the physicians would accept a financial proposition. In 62.2%, the physicians gave positive response to Q3, but they believed that only 21.1% of the other physicians would do so. More than 70% of the physicians claimed that they would talk to the physician of the center about this unethical action, and they assumed similar actions for only 12% of the other physicians.

*The Fifth Scenario*

A patient is treated for breast cancer in a university hospital. The patient needs annual mammography. The radiology center A is a governmental center and the radiology center B is a private one without insurance coverage, and the patient should pay the costs.

Almost 90% of the physicians claimed that ‘they would refer the patients to the best medical center regarding the quality and response (time) length based on the inspections (Q1), and 98% of them claimed that they would explain the reason for choosing that medical center to the patients (Q2). The physicians also supposed that nearly 76% of the other physicians had chosen the best medical center based on inspection (Q1), and only 48% of the physicians would explain the reason for choosing the medical center to the patients (Q2). Almost 96% of the physicians claimed that ‘notwithstanding the consideration of the patient’s advantage, they attempt to give the right selection to the patient’; however, they believed that only 45.6% of the other physicians would do so. More than half of the physicians stated that ‘they had considered the risks resulting from COI, and they had not referred the patient from the state medical centers to the private ones’. Nevertheless, the participants believed that less than one third of the other physicians might do so.

## Discussion

Conflict of interest can exist in the fields of treatment, education or research. Giving gifts is a common way to promote and develop use of products of manufacturing companies. Generally, in this study, the physicians considered their performance better than that of other physicians. This finding has been proven in other studies, and most physicians believed that others were more affected than themselves (Fickweiler et al., 2017). Many physicians argue that they will not be affected by the industry influence. However, studies indicate that gifts and hospitality can endanger the judgment considering medical knowledge and decisions on patient care (Coyle, 2002). 

In this research, it was clarified that more than 70% of the physicians had stated that they did not allow advertisement in their environment. Previous studies showed that most physicians did not perceive the difference between the promotion of a medicine and the scientific evidence related to the medicine (Fickweiler et al., 2017). At least one third of the physicians stated that they would accept a trip proposition from the medicine-promoting company; however, most of them claimed that the promotion had no effect on medicine prescription. A study conducted on American radiotherapists showed that 15% of physicians had a background of accepting the cost of a trip for participating in the congress, which is half of the amount found in our study (Halperin et al., 2004). The results of meta-analyses on similar studies indicated a relation between the pharmaceutical industry propositions and the effect on prescription behavior (Brax et al., 2017). Based on the ethical principles, any gift directing affecting the medicine prescription has to be avoided (Halperin et al., 2004).

Most of the physicians present in the study have agreed to participate in a congress, but only a half of the physicians have stated that they prescribed medicine based on the congress trainings and 90% of them stated that they would conduct the literature search about the medicine. Almost one fifth of the physicians stated that they became pessimistic about the promoted drug. According to the American Medical Association (AMA), all participants in a speech and continuous educational programs should ensure that the speech would not be inappropriately affected by the program sponsors. In fact, in any activity or program in which a pharmaceutical company is involved, 4the physicians should ensure that the program is not promotional, but rather educational (Halperin et al., 2004). The results of a systematic review indicated a relation between increasing prescribing rates, increased prescribing costs and low prescription quality and interactions with pharmaceutical companies (Fugh-Berman and Homedes, 2018). 

It appears that the solution for divulging the financial, vocational and personal relations in the researches is to create registrations with free national access. Therefore, the governing body should be responsible for monitoring organizations through evaluating the staff annually by the verification committees of national research and in the case of evidence of unethical practices, the research permit should be suspended (Bion et al., 2018). A recent study on assessing COI in the authors of textbooks has showed a considerable subset of biomedical authors is financially related to medical product companies. The findings of this study emphasize the importance of standard practices existing among authors involved in biomedical educational materials (Piper et al., 2018).

Almost one fifth of the physicians have stated that they would accept financial propositions for themselves, and 40% of them have claimed that they would accept it for dedication to the financially poor patients. Half of the physicians became suspicious of the finance-proposing company. Almost 80% of the physicians have stated prescription of a medicine after a comprehensive literature search, and have warned about unethical behavior. Previous studies indicated that direct payment was reported as 5% and 16% in two studies in Libya and Australia, respectively (Shahi et al., 2017). The American Medical Association states that the donation to the physicians should primarily address the patients’ benefit, and its amount should not be high. Furthermore, it has been recommended that cash donations not be accepted by the physicians (Halperin et al., 2004). It appears that direct financial gifts and drug samples should be prohibited and replaced with a system of vouchers for poor and low-income patients (Brennan et al., 2006).

Most physicians stated that they had referred the patients to centers with higher quality, and nearly 100% of the physicians stated that they would explain the reason of that choice. Almost all physicians gave the right of choice to the patients, and more than a half of the physicians had not referred the patient from a state center to a private center due to COI. Based on the codes of medical ethics, referral of patients between institutions and centers for more profit is unethical. The physicians’ commitment to humanity, putting the patients’ benefit in preference, practicing scientifically, and having no bias in medical decisions frequently expose them to COImost of which are financial. One of the most challenging conflicts results from the relation between the physicians with each other. 

In any occurrence of COI, there are two parties, the proposer and the acceptor of the gain. apparently, physicians, due to being subjected to receiving propositions from the companies seeking benefit, need to receive education during university and continuous education after initiating clinical work in order to be able to decide appropriately in COI situations (Halperin et al., 2004). Previous studies showed that education could influence the physicians’ attitude and practice (Shahi et al., 2017). In addition, it seems best that students not be subjected to the company promotions, and be trained by some courses for improvement of COI knowledge and its management (Bion et al., 2018). Moreover, increasing the sellers’ knowledge about immoral sales methods, enabling them to judge in unethical sales situations, and defining unethical selling are also important. A study to review and compare the intervention of COI education through entertainment-education and e-learning courses in the United States demonstrated that both methods significantly affected the improvement of the salespeople’s behavior (Miller, 2018). In addition, business ethics must be considered, which is a system of moral principles applied in the commercial world (BRONI, 2010). Furthermore, international prohibitory legislation and laws to punish the companies choosing unethical practice to promote their business can help to decrease COI.

In conclusion, this study showed that the physicians in the field of breast cancer were at the risk of COI. Although the existence of such a situation is not in itself contrary to ethical principles and professional conduct, the extent of its frequent occurrence indicates the need for special attention to this issue. The important finding of this study is the physicians’ different understanding of their colleagues’ behavior in COI situations compared to their own performance. In other words, even within the medical field, there is not enough trust in the proper functioning of doctors in dealing with a conflict of interest situation. Creation of effective changes in the teaching of learners and definition of post-graduation training courses may be an effective way to improve clinicians’ performance. Furthermore, establishing appropriate engagement with pharmaceutical companies, manufacturing and distribution companies, as well as conducting courses for identifying logical ethical codes are suitable for pharmaceutical and medical marketers, which can improve the overall situation. This action can reduce the likelihood of conflict of interest by transparency of industrial relations within the medical community.
